# Photoelectric and Thermoelectric Dual Modulation Via a Ternary Composite

**DOI:** 10.1002/gch2.201800077

**Published:** 2018-12-18

**Authors:** Sam‐Shajing Sun, Harold Odell Lee

**Affiliations:** ^1^ Center for Materials Research PhD Program in Materials Science and Engineering Norfolk State University 700 Park Avenue Norfolk VA 23504 USA

**Keywords:** dual conversion, dual modulation, photoelectric conversion, ternary composite, thermoelectric conversion

## Abstract

Materials for simultaneous photoelectric and thermo‐electric dual conversions and modulations, where photon can modulate the thermoelectric conversion, and temperature can modulate the photoelectric conversion, may find potential applications where light (including a laser) can remotely turn on, turn off, or modulate a thermoelectric generator, a cooler, or a temperature sensor, and vice versa, temperature (heating/cooling) can turn on, turn off, or modulate a photoelectric device such as a photo detector or a solar cell. Here, it is demonstrated that such simultaneous dual conversion or modulation can be achieved via a ternary composite, e.g., a poly‐3‐hexyl‐thiophene thin‐film doped with both phenyl‐C61‐butyric acid methyl ester and iodine. This finding may result in the development of lightweight, flexible shape, cost‐effective, renewable, environmentally friendly, biocompatible, and scalable materials, devices, and systems for clean energy harvestings (such as solar and waste heat dual energy harvesting) as well as light/heat dual‐sensing sensors, modulators, and controllers.

Renewable, clean, cost effective, and efficient photoelectric or thermoelectric energy conversions are essential for sustainable growth of civilization, as these technologies could potentially reduce human dependence on the burning of chemical fossil fuels. Organic photoelectric charge separation can be achieved via a weak donor/acceptor pair and weakly coupled composite, also called photoelectric doping,^1^ and that organic thermoelectric charge separation can be achieved via a strong donor/acceptor pair but weakly coupled composite, also called thermoelectric doping.^1^, ^2^, ^3^ Semi‐metal and high Seebeck effects have been reported in organic systems.^3^ A potential photoelectric/thermoelectric dual conversion or charge separation material may be achievable with both doping processes in one active composite. Here we demonstrate the feasibility of such dual conversion or modulation materials by utilizing a conjugated polymer poly‐3‐hexyl‐thiophene (P3HT) as the main charge carrier collection and transport media, phenyl‐C61‐butyric acid methyl ester (PCBM) as the photoelectric dopant, and iodine as the thermoelectric dopant.

**Figure** **1** exhibits molecular frontier orbital (highest occupied molecular orbital (HOMO)/lowest unoccupied molecular orbital (LUMO)) levels of the three components and the work functions of ITO and the aluminum electrodes. When the iodine/P3HT/PCBM ternary composite is excited with an energy matched photon, an electron at P3HT HOMO shall be excited to its LUMO first and then transfer quickly to and being trapped at the nearby LUMO of PCBM resulting in a positive hole (positive polaron) that is mobile and can be transported along the bulk P3HT medium. Similarly, when a heat is applied on the composite, an electron at P3HT HOMO can be excited to and being trapped at the nearby LUMO of the iodine resulting in a positive hole that is mobile and can be transported along the bulk P3HT medium. Therefore, a p‐type dual conversion or modulation material could be realized.

**Figure 1:**
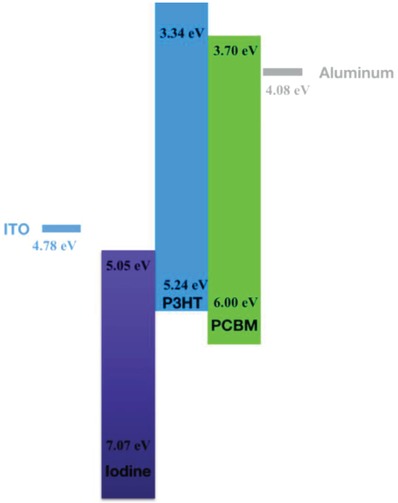
Scheme of frontier orbital levels of iodine, P3HT, PCBM, and Fermi levels of ITO and aluminum.

Iodine doped P3HT/PCBM composite has been studied before either to improve ambient condition conductivity of the P3HT/PCBM solar cell,^4^ or to enhance infrared radiation sensing of the P3HT/PCBM photo detector.^5^ Connecting or stacking a photoelectric cell on top of a thermoelectric cell together to harvest both light and heat (so called stacked or hybrid “Seebeck Solar Cell”) were also described,^6^, ^7^ but they were different from this study in that they were actually two separate materials and devices and there is no simultaneous thermoelectric and photoelectric dual functional switching or modulation in one material. Light induced Seebeck coefficient changes in certain semiconductors have also been reported.^8^, ^9^, ^10^ However, an intentionally doped ternary system for a targeted dual conversion and modulation has not yet been evaluated.

Materials electrical conductivity (σ) is defined in Equation (1) as

(1) σ=enμ

where *e* is the charge of the particle, *n* is the mobile charge carrier density, and μ is the charge mobility. A material's thermoelectric Seebeck coefficient (*S*) is defined and can be experimentally measured via Equation (2) (in units of µV K^−1^) as

(2) S=−ΔV/ΔT

The Seebeck coefficient is correlated to majority mobile charge carrier density *n* by Equation (3), 11 as

(3) $$S=\frac{8\pi^{2}k_{b}{}^{2}}{3eh^{2}}m^{*}T{\left(\frac{\pi }{3n}\right)}^{2/3}$$

where *k*_b_ is the Boltzmann constant, *e* is the carrier charge, *h* is Planck's constant, m* is the effective mass of the charge carrier, and *n* is the majority mobile charge carrier density. In our study, carrier density *n* is directly related to the photoelectric or thermoelectric doping induced positive polarons on P3HT, and that the carrier mobility μ is generally and directly correlated to materials solid state packing or film morphology. If the solid state packing or film morphology are the same or similar (such as within a narrow range of temperature change), then the conductivity can reflect doping induced carrier density changes.

Several film samples containing pristine P3HT, P3HT/iodine (five percent mol ratio of iodine in P3HT), and an iodine/P3HT/PCBM ternary composite (five percent mol ratio iodine/P3HT and a 1:1 weight ratio of P3HT/PCBM resulting in 5% iodine doped iodine/P3HT/PCBM composite processed from 1, 2‐dichlorobezene solution) was used in this demonstration due to our recent studies on doping level and/or doping ratio optimizations of such composite.^12^, ^13^ **Figure** **2** exhibits measured conductivities of the pristine P3HT and iodine doped P3HT thin films at room temperature in the dark (RT, 25 °C), at an elevated temperature in the dark (ET, 39 °C), at room temperature (RT + light) under a 50 mW cm^−2^ solar simulator light, and at an elevated 39 °C temperature (ET + light) under the same light. Conductivities of undoped or pristine P3HT at these same conditions were also evaluated, and the conductivity changes are negligible (generally less than 0.1%), and conductivity change due to possible morphology change can also be neglected. The conductivity of the iodine doped P3HT thin films increases when heated but when illuminated, no distinguishable change is seen which is expected since there is no photo acceptor to dissociate photoinduced excitons. **Figure** **3** exhibits measured conductivities of the ternary composite thin films under the same conditions as mentioned above. The conductivity increase from 2.47 × 10^−5^ S cm^−1^ at room temperature in the dark to 2.54 × 10^−5^ S cm^−1^ (about 2.8% increase) at elevated temperature in the dark can be attributed mainly to the thermoelectric charge separations between iodine and P3HT. Similarly, the conductivity increase from 2.47 × 10^−5^ S cm^−1^ at room temperature in the dark to 2.63 × 10^−5^ S cm^−1^ (about 7% increase) at room temperature under light illumination can be attributed mainly to the photoelectric charge separations between P3HT and PCBM. Finally, the conductivity increase from 2.47 × 10^−5^ S cm^−1^ at room temperature in the dark to 2.67 × 10^−5^ S cm^−1^ (about 8.5% increase) at elevated temperature under light illumination can be attributed to both the thermoelectric charge separations between P3HT and iodine, and the photoelectric charge separations between P3HT and PCBM, i.e., the conductivity data confirmed that both thermoelectric and photoelectric doping processes occurred in such ternary composite.

**Figure 2:**
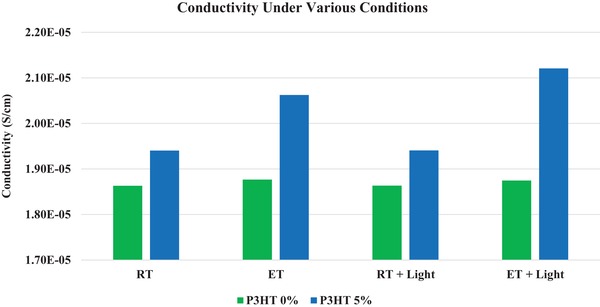
Measured conductivities of pristine P3HT and P3HT:iodine thin films at room temperature (RT, 25 °C) and an elevated temperature (ET, 39 °C) in the dark, at room temperature (RT + light), and an elevated temperature (ET + light) when illuminated with light.

**Figure 3:**
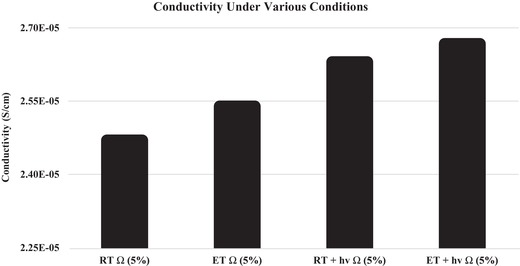
Measured conductivities of a I_2_:P3HT:PCBM ternary composite thin film at room temperature (RT, 25 °C) and an elevated temperature (ET, 39 °C) in the dark, at room temperature (RT + light), and an elevated temperature (ET + light) when illuminated with light.

In order to evaluate temperature modulation of the ternary composite film photoelectric conversion, **Figure** **4**a exhibits the measured photo *J–V* curves (under a 50 mW cm^−2^ solar simulator. Negative current density values are flipped to positive for better view of open circuit voltage *V*_oc_) at room temperature (25 °C) and at an elevated temperature (39 °C). Device data including open circuit voltage (*V*_oc_), short circuit current (*I*_sc_), the product of *V*_oc_ *J*_sc_, and power conversion efficiencies (PCEs) are listed in **Table** **1**. Since poly(3,4‐ethylenedioxythiophene):polystyrene sulfonate (PEDOT:PSS) and its similar systems are known to exhibit thermoelectric effects,^3^ therefore, no PEDOT or related hole collection layer were used in this device study. Additionally, without iodine doping, there is no difference between the photo *JV* curves of a P3HT:PCBM binary composite film at 25 and 39 °C. Therefore, the heating incurred photo *JV* curve changes shown in Figure 4a can be attributed mainly to mobile hole density increase in P3HT as a result of thermoelectric charge separation between iodine and P3HT. In order to evaluate the photo modulation of the thermoelectric conversion of the ternary composite film, Figure 4b exhibits the measured thermoelectric Seebeck coefficients of such ternary composite thin films under dark and under different light illumination intensities up to about 20 mW cm^−2^. The initial increase of Seebeck coefficient versus light intensity from dark to about 8 mW cm^−2^ could be attributed to an electron–photon coupling mechanism that are dominant at low light intensity or low charge density regime,^10^ and the decrease of the Seebeck coefficient passing the 8 mW cm^−2^ can be accounted by a dominant Seebeck versus mobile carrier density relationship expressed in Equation (3).^11^

**Figure 4:**
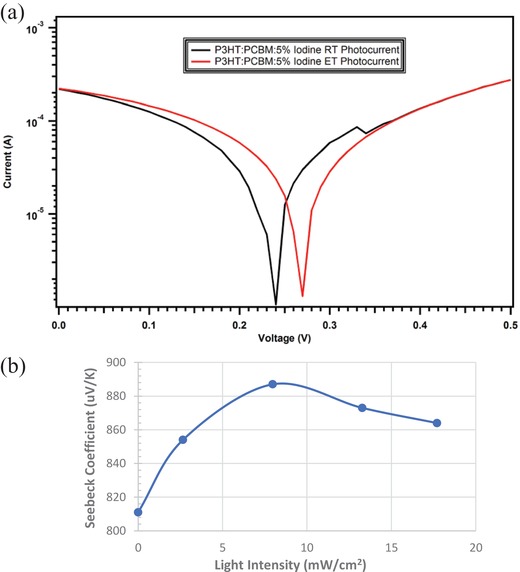
a) Temperature modulation of photo *JV* curves of a I_2_:P3HT:PCBM ternary composite thin film at room temperature (RT, 25 °C) versus an elevated temperature (ET, 39 °C). b) Light modulation of thermoelectric Seebeck coefficients of the same composite thin film at different light illumination intensities.

**Table 1:** Measured and calculated device parameters of a ITO/I_2_:P3HT:PCBM/Al photoelectric/thermoelectric dual modulation device at room temperature (RT, 25 °C) and at elevated temperature (ET, 39 °C)

	RT (25 °C)	ET (39 °C)	Change
*I*_sc_ [mA]	0.21958	0.22200	1.09%
*V*_oc_ [mV]	235	265	11.32%
*I*_sc_ × *V*_oc_ [mW]	51.60	58.83	12.29%
PCE [%]	0.677	0.832	18.63%

Our preliminary experimental data demonstrates that photon can be used to effectively modulate the thermoelectric conversions of a material, and temperature can simultaneously modulate the photoelectric conversions of the same materials if the material is doped with both a photoelectric dopant as well as a thermoelectric dopant. In this demonstration, the change of thermoelectric Seebeck coefficient between dark and highest photo modulated Seebeck is about 9.4%. With further and systematic studies and optimizations, it is feasible that light could more effectively modulate the thermoelectric conversion of the material.

Our preliminary experimental results and data confirmed the feasibility of a photoelectric/thermoelectric dual conversion modulator/switching material and device. However, materials, processing, fabrications, and device parameters need to be systematically investigated and optimized in order to fit a particular application need.

Potential applications of such photoelectric/thermoelectric dual conversion and dual function modulator/switching materials and devices may include, but may not limited to, an application where light (including a laser beam) can remotely turn on, turn off, or modulate a thermoelectric generator, a thermoelectric cooler, or a temperature sensor. Vice versa, the temperature (heating or cooling) can turn on, turn off, or modulate a photoelectric device such as a solar cell or a photo/radiation detector. The potential impacts of this work may result in the systematic development of lightweight, flexible shape, cost effective, renewable, environmental friendly, biocompatible, and scalable materials, devices, and systems for clean and renewable energy conversions (such as solar and waste heat energy conversions) and light/heat multiinput signal sensing and modulation.

## Conflict of Interest

The authors declare no conflict of interest.
